# Burden of childhood diseases and malnutrition in a semi-urban slum in southern India

**DOI:** 10.1186/1471-2458-13-87

**Published:** 2013-01-30

**Authors:** Rajiv Sarkar, Prabhu Sivarathinaswamy, Bhuvaneshwari Thangaraj, Kulandaipalayam Natarajan Chella Sindhu, Sitara Swarna Rao Ajjampur, Jayaprakash Muliyil, Vinohar Balraj, Elena N Naumova, Honorine Ward, Gagandeep Kang

**Affiliations:** 1 Department of Gastrointestinal Sciences, Christian Medical College, Vellore, Tamil Nadu, 632004, India; 2 Community Health Department, Christian Medical College, Vellore, Tamil Nadu, 632002, India; 3 Department of Civil and Environmental Engineering, Tufts University School of Engineering, Medford, MA, 02155, USA; 4 Division of Geographic Medicine and Infectious Diseases, Tufts Medical Center, Tufts University School of Medicine, Boston, MA, 02111, USA

**Keywords:** Children, Morbidity, Incidence, Slum, Longitudinal study, India

## Abstract

**Background:**

India has seen rapid unorganized urbanization in the past few decades. However, the burden of childhood diseases and malnutrition in such populations is difficult to quantify. The morbidity experience of children living in semi-urban slums of a southern Indian city is described.

**Methods:**

A total of 176 children were recruited pre-weaning from four geographically adjacent, semi-urban slums located in the western outskirts of Vellore, Tamil Nadu for a study on water safety and enteric infections and received either bottled or municipal drinking water based on their area of residence. Children were visited weekly at home and had anthropometry measured monthly until their second birthday.

**Results:**

A total of 3932 episodes of illness were recorded during the follow-up period, resulting in an incidence of 12.5 illnesses/child-year, with more illness during infancy than in the second year of life. Respiratory, mostly upper respiratory infections, and gastrointestinal illnesses were most common. Approximately one-third of children were stunted at two years of age, and two-thirds had at least one episode of growth failure during the two years of follow up. No differences in morbidity were seen between children who received bottled and municipal water.

**Conclusions:**

Our study found a high burden of childhood diseases and malnutrition among urban slum dwellers in southern India. Frequent illnesses may adversely impact children’s health and development, besides placing an additional burden on families who need to seek healthcare and find resources to manage illness.

## Background

The UN Millennium Development Goal (MDG) 4 aims to reduce the global under-five mortality rates by two-thirds between the years 1990 and 2015 [1]. Although mortality rates have declined globally by about 28% between 1990 and 2008, about 8.8 million children still die each year before they reach their fifth birthday, with 99% of these deaths occurring in low and middle income countries [2]. India is the largest contributor, accounting for about one-fifth of the global under-five deaths, totaling approximately 1.8 million child deaths annually [3].

In India, between the years 1991 and 2001, a total of 14.3 million people migrated from rural to urban areas. In cities with populations of over a million, nearly one-fourth of the urban population resides in slums [4]. This rapid increase in slum populations poses problems such as poor housing conditions, overcrowding, lack of potable drinking water and sanitation facilities [5], which have a profound effect on human health. People living in slums have a wide range of communicable and non-communicable diseases [6].

Despite this, the magnitude, distribution and risk factors for the majority of diseases in slum populations are yet to be quantified in many developing countries. In most instances, the information on disease burden and mortality is based on clinic, hospital or national mortality registry data, which represent only the “tip of the iceberg” [7]. Long-term, prospective, population-based surveillance data is required to adequately assess the burden of acute and chronic diseases in such populations and to characterize their determinants. The data obtained through these prospective studies can be used for planning the distribution and utilization of available healthcare resources, and also to develop effective disease control measures.

Here, we present the morbidity experience of children living in semi-urban slums of Vellore in southern India, who participated in a quasi-experimental study on the effect of bottled drinking water on transmission of cryptosporidial infections. A previous birth cohort study conducted between 2002 and 2006 in the same locality had reported a high communicable disease burden among infants and children [8,9]. Widespread contamination of the drinking water supply has also been documented previously [10]. The quasi-experimental design employed in this study allowed us to ascertain whether or not the morbidity patterns differed in children drinking bottled water, in addition to capturing the long-term trends in childhood diseases in this population.

## Methods

### Study area and population

The study was conducted in Ramnaickapalayam, Chinnallapuram, Kaspa and Vasanthapuram, four geographically adjacent, semi-urban slums located in the western outskirts of Vellore, Tamil Nadu, India, with a population of about 40,000, comprising of approximately 50% Hindu, 45% Muslim and 5% Christian households. A large proportion of households use firewood as their primary cooking fuel. “Beedi-work” (manual production of indigenous tobacco-based cigarette-like products) is the predominant occupation, followed by unskilled labor. The majority of household incomes depend on daily-wage earners, who do not have the benefit of a regular salary and other benefits such as pension or health insurance. The residents receive piped drinking water, supplied by the local municipality intermittently (at intervals of 2–28 days), which they collect and store in multiple wide-mouthed containers and mostly consume without further treatment [10]. During times of water scarcity, bore-wells located at different parts of the study area and water supplied by the Vellore Municipal Corporation through tank trucks serve as an alternative source of drinking water.

A government urban health centre (UHC), providing free health care to the residents, is located within the study area; and a government teaching hospital is located approximately five km away. Numerous private facilities, clinics, nursing homes or hospitals and traditional medicine and faith healers are also located in close proximity. The Christian Medical College (CMC), Vellore, a not-for-profit organization and its two outreach units – the Community Health and Development (CHAD) and the Low Cost Effective Care Unit (LCECU), are located within a few kilometers of the study area.

The UHC records document a birth rate of 15.3 live births per 1000 population per year and an infant mortality rate of 18.2 deaths per 1000 live-births per year for the years 2008–2011. As per the sample registration survey conducted by the Government of India, the birth and infant mortality rates for urban Tamil Nadu for the year 2010 is 15.8 live births per 1000 population and 22 deaths per 1000 live-births respectively [11].

### Study design

A quasi-experimental (non-randomized) study was conducted to determine whether or not a protected water supply could prevent or delay cryptosporidial infections in children under the age of two years. Children were recruited at birth or while they were still being exclusively breast-fed, and their families received either bottled (protected) or municipal (unprotected) drinking water based on their area of residence. The water availability and consumption patterns were, however, similar among residents living in each of these areas, as was the degree of environmental contamination.

The bottled drinking water was obtained from a commercial provider in 20-liter Polyethylene terephthalate (PET) bottles. It is sourced from an underground aquifer and purified by reverse osmosis followed by ultraviolet irradiation and ozonization. The commercial provider follows quality control procedures to ensure that the physical, chemical and microbiological properties of the bottled water meet the specifications of the Bureau of Indian Standards (http://www.bis.org.in/cert/REQUIREMENTSIS14543.htm) (personal communication from the commercial bottled water provider). Prior to start of the study, multiple water samples from the same bottled water provider were independently tested using standard techniques [12], and were found to be free of microbial contamination (presumptive and fecal coliforms).

Supply of bottled water commenced at least one week prior to the scheduled weaning date of the enrolled child or, in case of unscheduled weaning, as soon as the field team became aware of the event. For the purpose of this study, a child was considered to be weaned if he/she were given anything to eat or drink other than breast-milk, including water. Sufficient water was provided for the drinking water needs of the entire household, with households encouraged to call if a replacement was needed ahead of the biweekly scheduled delivery.

### Enrollment and data collection

Prior to enrollment, a door-to-door survey of houses in the study area was conducted to identify families with women in late pregnancy or with children who were being exclusively breast-fed. Those families intending to stay in the area for at least two years were approached by the field staff for consent and recruitment. Children with gross congenital anomalies or with birth weight <1500 gm were excluded. The study was approved by the Institutional Review Boards at the Christian Medical College, Vellore, India, and the Tufts Medical Center and Tufts University Health Sciences Campus, Boston, USA. Recruitment was consecutive and followed written informed consent by parents or legal guardians of the eligible children. Each child was followed up until he/she attained the age of two years. The study commenced in September 2008 and ended in April 2011.

Soon after recruitment, field workers visited the house to obtain baseline demographic details and information on water usage and storage, toileting and animal contact. Socio-economic status (SES) of the participating families was also ascertained using a modified version of the Kuppuswamy scale as described previously in the same locality [9]. This composite SES scale takes into account the educational and occupational level of the family, house ownership, total number of rooms in the house (excluding kitchen and bathroom), and household possessions. Based on the above parameters, families received a score ranging from 0–5. A score of <2 denoted low SES.

Household hygiene was measured using a structured questionnaire covering the aspects of water, food and personal hygiene, which has previously been validated and used in the same community [9,10]. For this analysis, the hygiene measurement of each child closest to their time of weaning was used. Based on responses to the questionnaire a scoring system was developed and the households were assigned a hygiene score ranging from 0–18. Families whose scores were at or above the upper tertile (≥12) were considered as those with good household hygiene.

Every week, the field staff visited the study household and interviewed the caregiver about diarrhea, vomiting, fever, cold/cough or any other illness experienced by the child during each day since the last visit, using a structured questionnaire. They also enquired about diarrheal and other illnesses in the family, and were trained to use standard definitions to identify common morbidities.

A physician-run clinic was also set up within the study area to provide basic health care to all children under the age of five years in the locality. Primary caregivers were encouraged to bring their sick children to the clinic, and those requiring hospitalization or specialized care were referred to the secondary (CHAD) or tertiary care hospital (CMC), as deemed necessary by the study physician. A record of all such visits was maintained by the field staff. If a child was taken to any other health-care facility for treatment, the physician recorded-diagnosis in the prescription or discharge summary, if available, was obtained by the field staff during their weekly home visits.

### Assessment of malnutrition

Monthly anthropometric (weight and length/height) measurements were also recorded for all study children. Malnutrition in children was assessed by computing the height-for age (HAZ), weight-for-height (WHZ) and weight-for-age (WAZ) z-scores, using the 2006 WHO child growth standards as the reference population [13]. Based on their z-scores, children were classified as stunted (HAZ<−2 SD), wasted (WHZ<−2 SD), underweight (WAZ<−2 SD) or normal. Any child who was found to be either wasted or underweight was further classified as undernourished. A child was considered to be early and persistently stunted if the HAZ score was <−2 SD at 6 months and remained so at 12, 18 and 24 months of age. Similarly, children who were either wasted (WHZ <−2 SD) or underweight (WAZ <−2 SD) at 6, 12, 18 and 24 months were classified as early and persistently undernourished.

### Classification of morbidities

The morbidity experiences of each child, as reported by the primary caregiver or obtained from the discharge summary during the weekly visits were classified into five broad categories - respiratory, gastrointestinal (GI), skin, other infections and non-infectious morbidities. GI illness was defined as diarrhea (3 or more loose watery stools over a 24 h period [14]) or vomiting lasting for more than 24 h. Respiratory illnesses were classified as either upper (URI) or lower respiratory tract infections (LRI). LRI included bronchitis or pneumonia as diagnosed by a physician, whereas URI included cough, cold and runny nose with and without concomitant symptoms. For URIs without any concomitant symptom, only those lasting for five or more days were considered for analysis. Skin lesions consisted mostly of rashes, but also included a wide variety of other conditions such as vesicles, pustules, cysts, ulcerations and excoriations. Other infections included infections of the eyes, ears or any other localized infection with or without fever. Non-infectious morbidities included non-specific swellings, surgical conditions such as hernia and phimosis, congenital diseases, injuries, insect bites and accidents. For GI morbidities, a new episode was defined as illness occurring at least 48 h after resolution of the previous episode. For all other illnesses, this time was taken as 72 h.

### Statistical analysis

Data were entered in duplicate using the Epi-Info 2002 (CDC, Atlanta, GA, USA) software and analyzed using STATA 10.1 for Windows (StataCorp, College Station, TX, USA) software. Comparison of baseline differences between the bottled and municipal water cohorts was done using *χ*^2^ test or Fisher’s exact test for categorical variables and using two-tailed *t*-tests or Wilcoxon rank sum tests for continuous variables, depending on the distribution of data. Incidence rates were calculated as the number of episodes divided by the child-years of follow-up. The total person-time at risk was calculated as total days under surveillance minus days of missing surveillance data (if ≥1 week). In order to account for multiple failures in a child, Poisson survival models with robust standard errors were fitted to calculate the incidence rates for different disease conditions.

## Results

### Recruitment and data collection

The house-to-house survey identified a total of 193 children who fulfilled the eligibility criteria and their families were asked to participate in the study. Of these, 176 (91.2%) agreed to participate and provided informed consent. Ninety (51.1%) households of these children received bottled water, whereas the households of 86 (48.9%) children continued with municipal water usage. The median [inter-quartile range (IQR)] age at the start of follow-up was 22 (12.5-56) days, which was comparable between the two cohorts (*P*=0.812).

**Figure 1:**
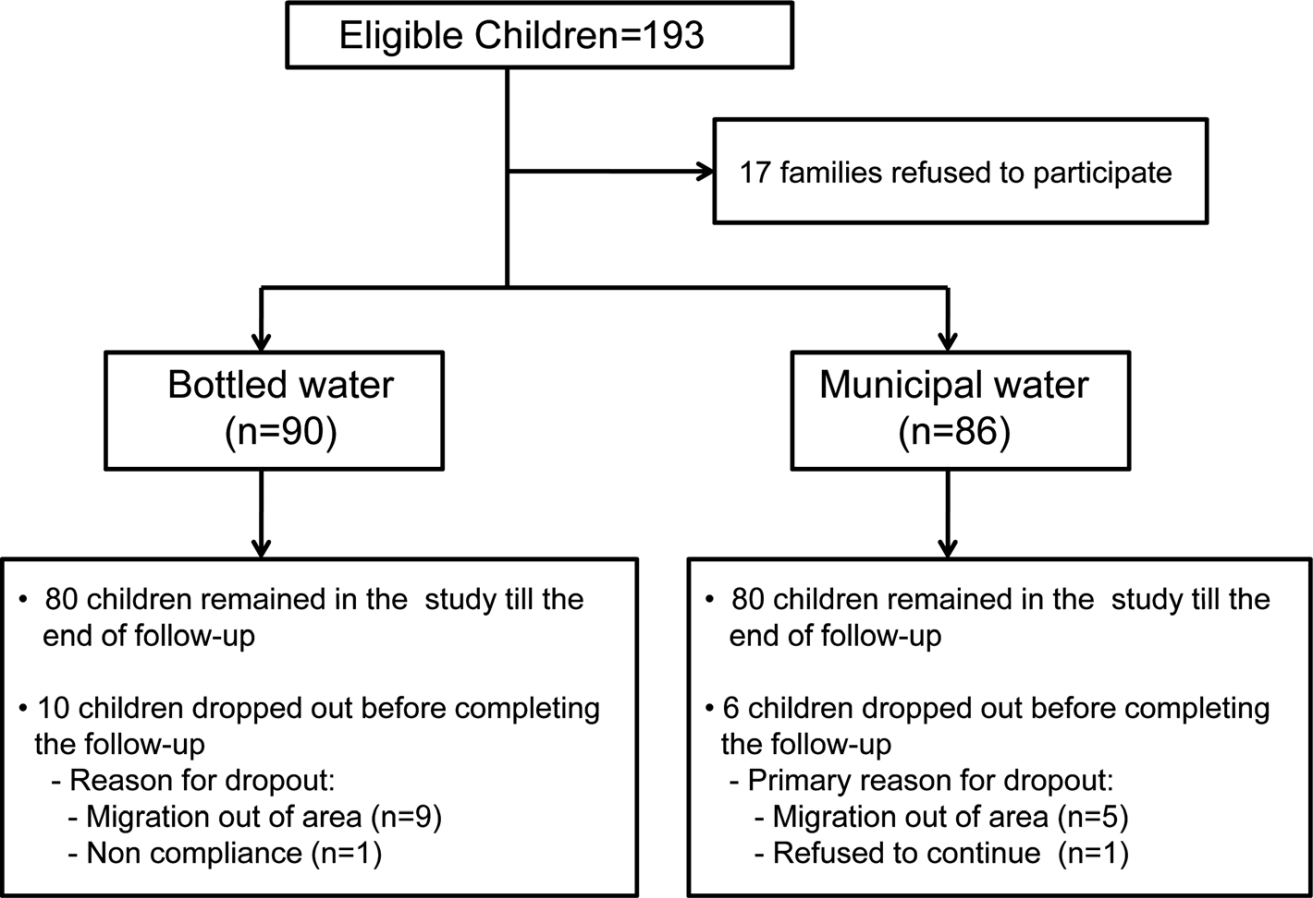
Flowchart of cohort recruitment and follow-up.

Of the 176 children enrolled into the study, 160 (90.9%) completed the two-year follow-up. The baseline socio-demographic characteristics of children who were lost to follow-up did not differ significantly from those who remained in the study until the end of follow-up (Table 1). The primary reason for loss to follow-up was migration out of the study area. There was no significant difference in the number of children lost to follow-up between those using bottled or municipal water (*P*=0.340). Children who dropped out, remained in the study for a median (IQR) period of 14.4 (5.7-17.4) months. The cohort recruitment and follow-up is outlined in Figure 1.

**Table 1:** Comparison of baseline characteristics between children who completed the follow-up and those who were lost to follow-up

	**Completed follow-up**	**Lost to follow-up**	***P*-value**
	**(n=160)**	**(n=16)**	
Male child	85 (53.1%)	10 (62.5%)	0.473 ^†^
Nuclear family	74 (46.3%)	9 (56.3%)	0.445 ^†^
Hindu religion	88 (55%)	9 (56.3%)	0.924 ^†^
Median (IQR) birth weight (in kg) *	2.9 (2.6-3.2)	2.9 (2.5-3.2)	0.790 ^‡^
Normal vaginal delivery	139 (86.9%)	15 (93.8%)	0.697 ^§^
Birth in a hospital/health-care facility	157 (98.1%)	16 (100%)	1.000 ^§^
Median (IQR) age (in months) at introduction of supplementary feeding	4.7 (3.6-5.7)	3.9 (2.5-5.4)	0.225 ^‡^
Median (IQR) family size	5 (4–7)	5 (3.5-7)	0.138 ^‡^
Presence of siblings	111 (69.4%)	8 (50%)	0.114 ^†^
Median (IQR) age of the mother (in years)	24 (22–26)	24 (21.5-26)	0.967 ^‡^
Median (IQR) years of completed maternal education	8 (3.5-10)	8 (6.5-10)	0.243 ^‡^
Median (IQR) years of completed education of the head of the household	5 (0–8)	6.5 (2.5-8)	0.277 ^‡^
Presence of cow in the house	14 (18.8%)	0 (0%)	0.370 ^§^
Presence of any animal in the house	47 (29.4%)	3 (18.8%)	0.562 ^§^
Living in a “kutcha” house	31 (19.4%)	3 (18.8%)	1.000 ^§^
Low socio-economic status	106 (66.3%)	11 (68.8%)	0.840 ^†^
Firewood as the primary cooking mode	83 (51.9%)	7 (43.8%)	0.535 ^†^
Presence of a functional toilet within the house ^¶^	98 (61.6%)	4 (66.7%)	1.000 ^§^
Good household hygiene	44 (27.5%)	3 (18.8%)	0.564 ^§^

* Data missing for 9 children; ^¶^ Data missing for 11 children.

Tests of significance: ^†^ χ^2^ test; ^§^ Fisher’s exact test; ^‡^ Wilcoxon rank sum test.

Eliminating periods where per-protocol follow up was not possible because the child was out of the study area, each child was followed-up for a median (IQR) of 22 (20.5-23) months, accounting for a total of 3764 child-months of follow-up. No significant difference in the number of monthly home visits was observed between children drinking bottled and municipal water (*P*=0.989).

### Baseline characteristics

Overall, children were born mostly into nuclear (83, 47.2%) or extended (75, 42.6%) families. The median (IQR) family size was 5 (4–7) members. The mean (SD) age of the mother at the time of birth of the child was 24.1 (3.4) years, and they had completed a median (IQR) of 8 (5–10) years of schooling. The majority of the children were Hindus (97, 55.1%), or Muslims (70, 39.8%), with only a small proportion of children (9, 5.1%) belonging to Christian households. Approximately two-thirds (117, 66.5%) of the children belonged to families classified as low socio-economic status. Firewood was the predominant cooking fuel for about half (90, 51.1%) of the recruited families.

More than half of the children were males (95, 54%) and over two-thirds (119, 67.6%) had older siblings. The median age at introduction of supplementary feeding was 4.6 (3.5-5.7) months. For the 167 (94.9%) children for whom birth weight data was available, mean (SD) weight at birth was 2.9 (0.5) kg, with 20 (12%) children classified as low birth weight (<2.5 kg).

Comparison of baseline characteristics between the bottled and municipal water cohorts (Table 2) revealed certain baseline differences. For example, children provided bottled water weaned earlier and had a smaller family size. Also, a greater proportion of families in the bottled water cohort belonged to the Hindu religion and had poorer household hygiene. On the other hand, significantly more families in the municipal water cohort used firewood as their primary cooking fuel. Compared to those in the municipal water cohort, mothers as well as the head of the households of children in the bottled water cohort had higher levels of education.

**Table 2:** Comparison of baseline characteristics between children in bottled and municipal water cohorts

	**Bottled water**	**Municipal water**	***P*-value**
	**(n=90)**	**(n=86)**	
Male child	47 (52.1%)	48 (55.8%)	0.633 ^†^
Nuclear family	43 (47.8%)	40 (46.5%)	0.866 ^†^
Hindu religion	64 (71.1%)	33 (38.4%)	<0.001 ^†^
Mean (SD) birth weight (in kg) *	2.9 (0.4)	2.9 (0.5)	0.786
Normal vaginal delivery	77 (85.6%)	77 (89.5%)	0.425 ^†^
Birth in a hospital/health-care facility	89 (98.9%)	84 (97.7%)	0.614 ^§^
Median (IQR) age (in months) at introduction of supplementary feeding	4.2 (3.1-5.1)	5.2 (3.8-6.0)	<0.001 ^‡^
Median (IQR) family size	5 (4–7)	6 (5–7)	0.043 ^‡^
Presence of siblings	56 (62.2%)	63 (73.3%)	0.118 ^†^
Mean (SD) age of the mother (in years)	24.3 (3.5)	24.0 (3.3)	0.664 ^Δ^
*Education of the head of the household:*			
No formal education	28 (31.1%)	37 (43.0%)	0.043 ^†^
Up to primary school (5 years)	14 (15.6%)	21 (24.4%)	
Middle School (6–8 years)	24 (26.7%)	16 (18.6%)	
High school and above (>8 years)	24 (26.7%)	12 (14.0%)	
*Education of the mother:*			
No formal education	13 (14.4%)	26 (30.2%)	0.019 ^†^
Up to primary school (5 years)	11 (12.2%)	16 (18.6%)	
Middle School (6–8 years)	26 (28.9%)	20 (23.3%)	
High school and above (>8 years)	40 (44.4%)	24 (27.9%)	
Presence of cow in the house	6 (6.7%)	8 (9.3%)	0.518 ^†^
Presence of any animal in the house	22 (24.4%)	28 (32.6%)	0.233 ^†^
Living in a “kutcha” house	22 (24.4%)	12 (13.9%)	0.078 ^†^
Low socio-economic status	65 (72.2%)	52 (60.5%)	0.099 ^†^
Firewood as the primary cooking mode	39 (43.3%)	51 (59.3%)	0.034 ^†^
Presence of a functional toilet within the house ^¶^	52 (62.7%)	50 (61.0%)	0.825 ^†^
Good household hygiene	12 (13.3%)	35 (40.7%)	<0.001 ^†^

* Data missing for 9 children; ^¶^ Data missing for 11 children.

Tests of significance: ^†^ χ^2^ test; ^§^ Fisher’s exact test; ^‡^ Wilcoxon rank sum test; ^Δ^ two-tailed *t*-test.

### Malnutrition among children in the bottled and municipal water cohorts

**Figure 2:**
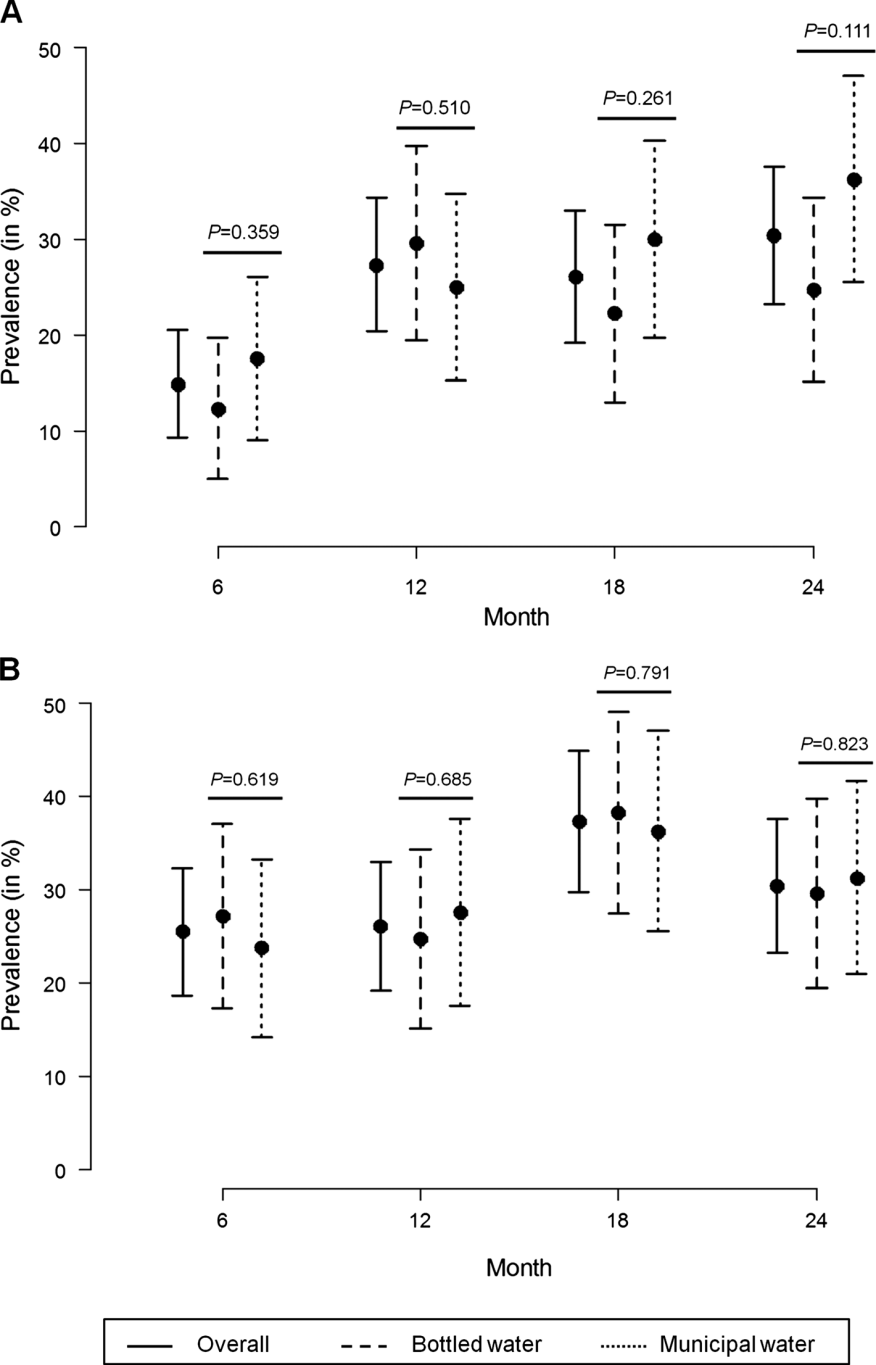
**Prevalence (with 95%CI) of (A) stunting and (B) undernutrition among the study children at 6, 12, 18 and 24 months of age.** The *P*-values represent the results of the comparison of prevalence of nutritional parameters between children in the bottled and municipal water cohorts.

Complete anthropometric data were available for 161 (91.5%) of the 176 children under observation. Figure 2 (A and B) presents the proportion (with 95% CI) of stunted and undernourished (wasted and/or underweight) children at the four different time points (6, 12, 18 and 24 months of age). Stunting was observed to increase progressively from 14.9% at 6 months to 30.4% at 24 months (Figure 2A). On the other hand, prevalence of undernutrition showed an increasing trend between 6 to 18 months of age, decreasing thereafter at 24 months (Figure 2B). The proportions of stunted and undernourished children were comparable between the bottled and municipal water cohorts at each of the four time points.

Twelve (7.5%) children were found to have early and persistent stunting, whereas 21 (13%) were persistently undernourished. Seventy (40.5%) and 85 (52.8%) children were found to be stunted and undernourished respectively in at least one of the four observations. A total of 101 (62.7%) children experienced one or more growth failures (stunted/wasted/underweight) during their follow-up period. When the nutritional parameters were compared between male and female children, no gender differences in the prevalence of either stunting or undernourishment was noticed at any of the four time points.

### Morbidity and mortality among children in the bottled and municipal water cohorts

A total of 3932 episodes of illnesses were recorded during the follow-up period, resulting in an incidence (95% CI) of 12.5 (11.9-13.2) episodes of illness/child-year. There was no significant difference in the overall morbidity pattern between children in the bottled and municipal water cohorts (12.1 episodes/child-year vs. 13.0 episodes/child-year, *P*=0.191). All except one child reported one or more episodes of illness during the follow-up period.

Children had more illnesses during infancy (13.8 episodes/child-year) than during the subsequent year (11.0 episodes/child-year), irrespective of whether they belonged to the municipal (14.2 vs. 11.6 episodes/child-year) or bottled water (13.5 vs. 10.4 episodes/child-year) cohort (Table 3).

**Table 3:** Incidence rates of morbidities and proportion of clinic visits and hospitalizations among children in bottled and municipal water cohorts

	**1st year**				**2nd year**			
	**Overall**	**Bottled water**	**Municipal water**	*P-*value *	**Overall**	**Bottled water**	**Municipal water**	*P-*value *
	**(n=176)**	**(n=90)**	**(n=86)**		**(n=170)**	**(n=86)**	**(n=84)**	
**All-cause morbidities:**								
No. of episodes	2331	1151	1180	---	1601	767	834	---
Rate of episodes/child-year (95% CI)	13.8 (13.1-14.7)	13.5 (12.4-14.8)	14.2 (13.2-15.3)	0.433	11.0 (10.3-11.8)	10.4 (9.3-11.7)	11.6 (10.7-12.6)	0.135
Episodes resulting in clinic visit: Number (% of total illness episodes)	1700 (72.9%)	847 (73.6%)	853 (72.3%)	0.480	1089 (68.0%)	515 (67.1%)	574 (68.8%)	0.472
Episodes resulting in hospitalization: Number (% of total illness episodes)	60 (2.6%)	39 (3.4%)	21 (1.8%)	0.014	26 (1.6%)	14 (1.8%)	12 (1.4%)	0.541
**Respiratory morbidities:**								
No. of episodes	1506	693	813	---	860	388	472	---
Rate of episodes/child-year (95% CI)	8.9 (8.5-9.4)	8.1 (7.5-8.8)	9.8 (9.1-10.5)	0.001	5.9 (5.5-6.4)	5.3 (4.7-5.9)	6.6 (6.0-7.2)	0.003
Episodes resulting in clinic visit: Number (%of total illness episodes)	1299 (86.2%)	612 (88.3%)	687 (84.5%)	0.032	733 (85.2%)	333 (85.8%)	400 (84.7%)	0.657
Episodes resulting in hospitalization: Number (% of total illness episodes)	32 (2.1%)	18 (2.6%)	14 (1.7%)	0.240	13 (1.5%)	9 (2.3%)	4 (0.85%)	0.078
**GI morbidities:**								
No. of episodes	668	367	301	---	384	184	200	---
Rate of episodes/child-year (95% CI)	4.0 (3.5-4.5)	4.3 (3.6-5.2)	3.6 (3.0-4.3)	0.162	2.6 (2.3-3.1)	2.5 (2.0-3.2)	2.8 (2.3-3.4)	0.483
Episodes resulting in clinic visit: Number (% of total illness episodes)	331 (49.6%)	197 (53.7%)	134 (44.5%)	0.019	197 (51.3%)	96 (52.2%)	101 (50.5%)	0.743
Episodes resulting in hospitalization: Number (% of total illness episodes)	18 (2.7%)	12 (3.3%)	6 (2.0%)	0.311	11 (2.9%)	5 (2.7%)	6 (3.0%)	0.868
**Skin lesions:**								
No. of episodes	86	47	39	---	176	96	80	---
Rate of episodes/child-year (95% CI)	0.51 (0.38-0.70)	0.55 (0.36-0.89)	0.47 (0.33-0.70)	0.574	1.2 (0.99-1.5)	1.3 (0.99-1.8)	1.1 (0.8-1.5)	0.449
Episodes resulting in clinic visit: Number (% of total illness episodes)	34 (39.5%)	14 (29.8%)	20 (51.3%)	0.042	83 (47.2%)	45 (46.9%)	38 (47.5%)	0.934
Episodes resulting in hospitalization: Number (% of total illness episodes)	4 (4.7%)	4 (8.5%)	0 (0%)	0.062	1 (0.57%)	0 (0%)	1 (1.3%)	0.272
**Other infections:**								
No. of episodes	47	26	21	---	88	54	34	---
Rate of episodes/child-year (95% CI)	0.28 (0.21-0.38)	0.31 (0.21-0.47)	0.25 (0.16-0.42)	0.537	0.61 (0.46-0.81)	0.73 (0.51-1.1)	0.47 (0.32-0.73)	0.118
Episodes resulting in clinic visit: Number (% of total illness episodes)	23 (52.3%)	13 (50%)	10 (47.6%)	0.871	34 (38.6%)	23 (42.6%)	11 (32.4%)	0.337
Episodes resulting in hospitalization: Number (% of total illness episodes)	5 (10.6%)	4 (15.4%)	1 (4.8%)	0.240	1 (1.1%)	0 (0%)	1 (2.9%)	0.205
**Non-infectious morbidities:**								
No. of episodes	24	18	6	---	93	45	48	---
Rate of episodes/child-year (95% CI)	0.14 (0.09-0.22)	0.21 (0.13-0.35)	0.07 (0.03-0.23)	0.038	0.64 (0.51-0.81)	0.61 (0.44-0.87)	0.67 (0.50-0.92)	0.700
Episodes resulting in clinic visit: Number (% of total illness episodes)	13 (54.2%)	11 (61.1%)	2 (33.3%)	0.237	42 (45.2%)	18 (40%)	24 (50%)	0.333
Episodes resulting in hospitalization: Number (% of total illness episodes)	1 (4.2%)	1 (5.6%)	0 (0%)	0.555	0 (0%)	0 (0%)	0 (0%)	---

*P-values obtained by comparing the rates in bottled and municipal cohorts using Poisson survival models with robust standard errors.

**Figure 3:**
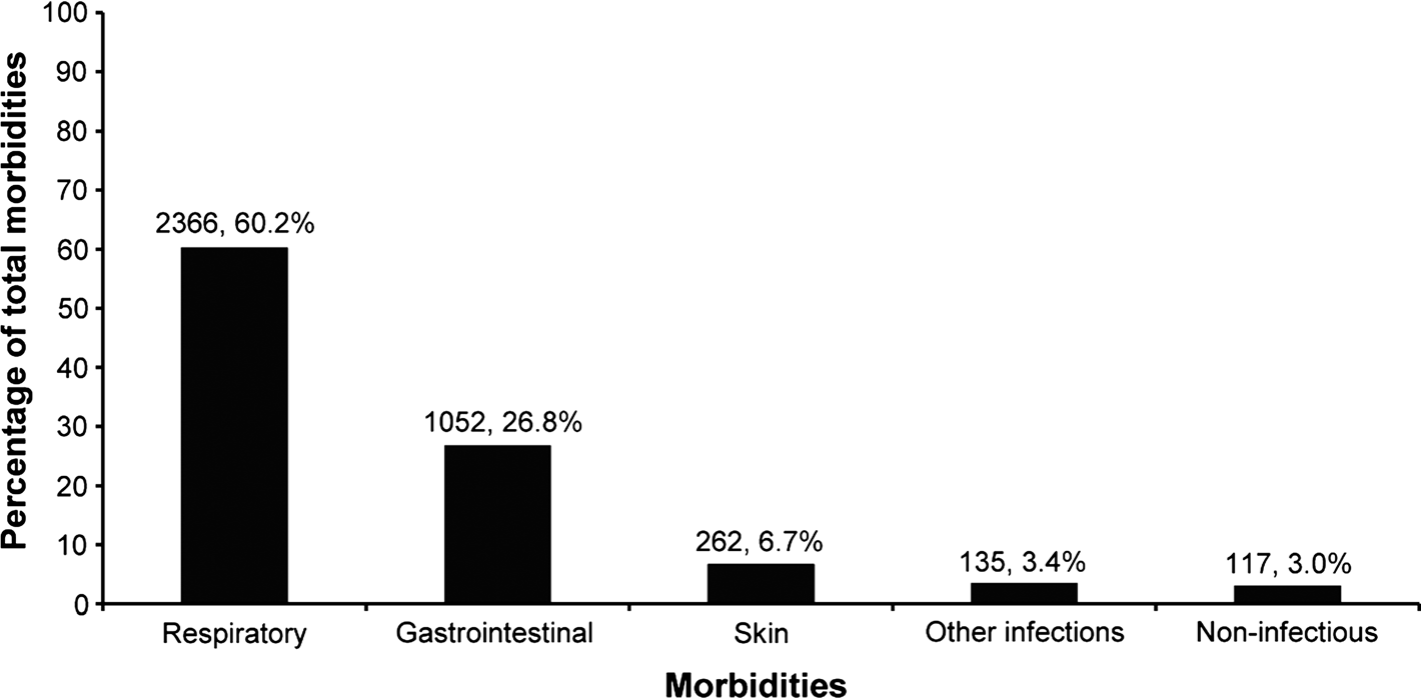
The contribution of different illness categories towards the overall morbidity experience of the study children.

Respiratory morbidity was the most common (Figure 3), comprising 60.2% of all reported morbidities. The incidence (95% CI) of respiratory morbidities was calculated at 7.5 (7.2-7.9) episodes/child-year. Children in the municipal water cohort reported significantly more respiratory morbidities (8.3 episodes/child-year) than those in the bottled water cohort (6.8 episodes/child-year, *P*<0.001). This difference persisted even after adjusting for the effect of potential confounding variables (Table 4). Almost all respiratory morbidities (98%) were URIs. The median (IQR) duration of an episode of respiratory illness was 10 (6–21) days, since all uncomplicated URIs of less than 5 days were excluded. On an average, children had an additional 3 episodes of respiratory illness in infancy than during the subsequent year. This trend was similar in children who received bottled and municipal water (Table 3).

**Table 4:** Results of unadjusted and adjusted analysis to assess the effect of bottled drinking water on the incidence rates of different morbidities among the study children

	**1st year (n=176)**				**2nd year (n=170)**			
	**Unadjusted Analysis**^¶^		Adjusted Analysis *^¶^		**Unadjusted Analysis**^¶^		Adjusted Analysis *^¶^	
**Morbidities**	**Rate Ratio (95%CI)**	***P*-value**	**Rate Ratio (95%CI)**	***P*-value**	**Rate Ratio (95%CI)**	***P*-value**	**Rate Ratio (95%CI)**	***P*-value**
Respiratory	0.83 (0.75-0.92)	0.001	0.85 (0.75-0.96)	0.007	0.80 (0.69-0.93)	0.003	0.81 (0.68-0.96)	0.015
Gastrointestinal	1.19 (0.93-1.53)	0.162	1.19 (0.87-1.63)	0.271	0.90 (0.66-1.21)	0.483	0.87 (0.58-1.31)	0.507
Skin	1.18 (0.66-2.09)	0.574	1.52 (0.79-2.95)	0.211	1.17 (0.78-1.76)	0.449	0.97 (0.63-1.49)	0.878
Other infections	1.21 (0.66-2.22)	0.537	0.98 (0.51-1.88)	0.949	1.55 (0.89-2.69)	0.118	1.27 (0.65-2.50)	0.480
Non-infectious	2.93 (1.06-8.11)	0.038	2.80 (0.80-9.80)	0.107	0.91 (0.58-1.44)	0.700	0.90 (0.53-1.51)	0.687

* Adjusted for: family size, socioeconomic status, use of firewood as the primary cooking fuel, maternal education, presence of toilet at home, Hindu religion, duration of exclusive breast feeding (in months), persistent stunting and household hygiene.

^¶^ Rate ratios and *P*-values calculated using Poisson survival models with robust standard errors.

GI morbidities accounted for 26.8% of all reported illnesses (Figure 3), with an incidence (95% CI) of 3.4 (3.0-3.8) episodes of GI illness/child-year. The majority of GI morbidities (807 episodes, 76.7%) were diarrheal episodes (with or without vomiting), followed by vomiting without diarrhea (243 episodes, 23.1%). The overall diarrheal incidence (95% CI) was 2.6 (2.3-2.9) episodes/child-year, whereas that of vomiting was 0.77 (0.63-0.97) episodes/child-year of follow-up.

The incidence of GI morbidities decreased from 4.0 episodes/child-year during infancy to 2.6 episodes/child-year during the second year of their life. No difference in the incidence of GI morbidities was noticed between children in the bottled and municipal drinking water cohorts (3.5 episodes/child-year vs. 3.2 episodes/child-year, *P*=0.549), even after adjusting for potential confounders (Table 4). The median (IQR) duration of an episode of GI morbidity was 3 (2–4) days.

When the diarrheal incidence rates were considered separately for the first two years of life, it was found to decrease from 3.1 episodes/child-year during infancy to 2 episodes/child-year during the second year. This decrease was more noticeable among children drinking bottled water (from 3.3 episodes to 1.8 episodes/child-year) than those drinking municipal water (from 2.8 episodes to 2.2 episodes/child-year), although the overall rates were comparable between children in the two cohorts (2.6 episodes/child-year and 2.5 episodes/child-year respectively, *P*=0.654).

Incidences of other illnesses ranged from 0.83-0.37 episodes/child-year and were equally distributed between the bottled and municipal water cohorts except for non-infectious morbidities, which was found to be significantly higher during infancy among children using bottled water (0.21 episodes/child-year vs. 0.07 episodes/child year, *P*=0.038). However, this difference was not statistically significant in the adjusted analysis (Table 4). In general, the incidence of skin, and other infectious and non-infectious diseases were higher among children during the second year of their life (Table 3). No deaths were reported among the study children.

A large proportion of morbidities (2789/3932, 70.9%) resulted in clinic or hospital outpatient visits. Among all morbidities reported, healthcare (clinic visit and/or hospitalization) was sought more frequently for respiratory illnesses (2077/2366, 87.8%), followed by gastrointestinal (557/1052, 52.9%) and non-infectious (56/117, 47.9%) morbidities (Table 3). Overall, 2.2% (86/3932) of all morbidities required hospitalization, at a rate of 0.3 (0.2-0.4) hospitalizations/child-year. A greater proportion of gastrointestinal (29/1052, 2.8%) and other infectious diseases (6/135, 4.4%) required hospitalization than respiratory (45/2366, 1.9%), skin (5/262, 1.9%) and non-infectious (1/117, 0.8%) diseases.

## Discussion

Respiratory and diarrheal diseases are two major causes of morbidity and mortality among children residing in the urban slums of India [8,15] and other developing countries [16,17]. In our study, respiratory and gastrointestinal illnesses together accounted for 87% of all childhood morbidities. The overall morbidity burden was high with children suffering from an average of 12.5 episodes of illnesses/year during the first two years of their lives. The findings of our study are comparable to the morbidity experience of children participating in a previous birth cohort study on rotavirus infections between the years 2002 and 2006 in the same slum population (Table 5), where they had an average of 11 episodes of different illnesses/year during the first three years of their lives [8]. However, another study conducted in 1965 among residents of 3 localities in Vellore town had reported a much higher morbidity burden in infants and children, with infants experiencing an average of 17.4 morbidities/child-year of observation (Table 5) [18]. Together, these findings suggest a decreasing trend in the burden of childhood diseases over the years in this region, possibly due to better access to health care, although the overall disease burden still remains high.

**Table 5:** Selected longitudinal studies on morbidities of children residing in urban slums of India and other developing countries, sorted chronologically

**Location**	**Year(s)**	**Frequency of surveillance**	**Sample size**	**Age group(s)**	**Measures of morbidity**		**Ref.**
					**Type of illness**	**Frequency**	
Vellore, India	1965-1967	Twice weekly	110 families	0-1 year	Total morbidity	Incidence - 17.4 per child-year	[18]
					Respiratory	Incidence - 6.9/child-year	
					Diarrhea	Incidence - 4.7/child-year	
				1-2 years	Total morbidity	Incidence - 15.4 per child-year	
					Respiratory	Incidence −6.3/child-year	
					Diarrhea	Incidence - 3.4/child-year	
Lima, Peru	1982-1984	Thrice weekly	153	0-11 months	Diarrhea	Incidence - 9.8/child-year	[19]
					LRI	Incidence - 1.0/child-year	
					Otitis Media	Incidence - 0.8/child-year	
Manila, Philippines	1985-1987	Weekly	1978	<5 years	ARI	Incidence - 6.1/child-year	[20]
					LRI	Incidence - 0.5/child-year	
Bangkok, Thailand	1986-1987	Twice weekly	674	<5 years	ARI	Incidence - 11.2/child year	[21]
					mild URI	Incidence - 9/child-year	
					moderate to severe URI	Incidence - 2.2/child-year	
					LRI	Incidence - 0.07/child-year	
Rio de Janeiro, Brazil	1987-1989	Weekly	229	<2 years	ARI	Incidence - 4.5/100 child-weeks	[22]
					LRI	Incidence - 0.8/100 child-weeks	
Salvador, Brazil	1989-1990	Alternate days	84	<40 months	Diarrhea	Incidence - 2.8/child-year	[23]
Nairobi, Kenya	1989-1990	Once every 3 days	920	3-37 months	Diarrhea	Incidence - 3.5/child-year	[24]
Fortaleza, Brazil	1989-1993	Weekly	71	6-21 months	ARI	Incidence - 10/child-year	[16]
					Diarrhea	Incidence - 7/child-year	
					Infective dermatitis	Incidence - 1/child-year	
					Respiratory	Prevalence - 15%	
Agartala, India	1992-1993	Twice weekly	400	<5 years	ARI	Mean monthly incidence - 23/100 children	[25]
					Diarrhea	Prevalence - 6.3%	
					Skin disorders	Prevalence - 4.5%	
Dhaka, Bangladesh	1999-2002	Alternate days	289	2-5 years	Diarrhea	Incidence - 1.8/child-year	[26]
Vellore, India	2002-2006	Twice weekly	452	<1 year	Total morbidity	Incidence - 12.0/child-year	[8,9]
					Respiratory	Incidence - 7.4/child-year	
					GI	Incidence - 3.6/child-year	
				1-2 years	Total morbidity	Incidence - 11.3/child-year	
					Respiratory	Incidence - 7.1/child-year	
					GI	Incidence - 1.6/child-year	
Kiberia, Kenya	2006-2008	Fortnightly (3 days recall)	5794	<5 years	ARI	Incidence - 0.5/child-year *	[27]
					Diarrhea	Incidence - 0.7/child-year *	
					Fever	Incidence - 0.09/child-year *	

* Data based on number of clinic visits.

ARI – Acute respiratory infections; URI- Upper respiratory tract infections; LRI – Lower respiratory tract infections; GI – Gastrointestinal morbidities.

Respiratory illnesses were the commonest cause of morbidity among children in our study, which is in concordance with findings of the previous birth cohort study, in which respiratory illnesses contributed to more than 50% of the total disease burden [8]. A higher burden of respiratory illnesses has also been reported from longitudinal studies conducted among underprivileged children in Peru [19] and Brazil [16]. The incidence of acute respiratory infections (ARI) among children in developing countries is estimated to range between 12.7 and 16.8 episodes/100 child-weeks [28], which translates to an annual incidence rate of 6.6-8.8 episodes per child. It is also possible that the high incidence of respiratory illnesses in our study is partly due to the active follow up.

The majority of respiratory illnesses reported in our study were upper respiratory infections, which is in agreement with other prospective studies from developing countries [20-22,29]. The incidence of respiratory morbidities was highest during infancy, decreasing thereafter, which differed from our previous birth cohort study where the incidence of respiratory infections was comparable across the three years of follow-up [8]. A decrease in the incidence of acute respiratory infections (ARI) with increasing age has also been noticed among children in rural Kenya [30]. In general, the rates of ARI seem to peak among infants aged 6–11 months [18,21,22], although higher rates in older children have also been observed [31]. An interesting finding was the significantly higher respiratory morbidity in the municipal drinking water cohort, which is possibly due to the greater use of firewood as the primary cooking fuel among these families (Table 2). The relationship between indoor air pollution from use of solid fuels such as firewood and respiratory illnesses in children has previously been documented [32-34].

GI morbidities were the next most common cause of morbidities among children in our study, almost all of which were episodes of either diarrhea or vomiting (without diarrhea). Taken alone, diarrhea accounted for approximately one-fifth of the overall disease burden. The diarrheal incidence in our study closely resembles the findings from a community-based longitudinal study among children under the age of five years residing in two urban slums of Brazil, where an overall incidence of 2.8 episodes of diarrhea/child/year was reported [23]. However, in another longitudinal study among rural Bangladeshi children, an incidence rate of 1.8 diarrheal episodes/child-year was reported [26], possibly due to the relatively older age (2–5 years) of the children. The morbidity estimates from the global burden of diarrheal disease study among under-five children in developing countries are, however, much higher at 3.2 episodes/child-year [35]. The temporal and regional difference in the diarrheal incidence (Table 5) thus underscores the need for contemporaneous, region-specific data to accurately estimate the diarrheal disease burden.

Although it was expected that the incidence of GI morbidities will be lower among children drinking bottled water, none of the illness categories other than respiratory illnesses were found to have lower rates. The apparent lack of negative association between bottled water and GI illnesses in our study could be due to contamination of drinking water at the point-of-use. In a study among peri-urban households in Lima, Peru, boiling of water failed to ensure its safety at the end-user level because of contaminated containers and poor domestic hygiene [36]. In the same study, boiled water was found to be more contaminated when served in a drinking cup than when taken directly from a container [36]. Most households in our study poured their drinking water into small, wide-mouthed containers prior to consumption, which were cleaned with locally available water sources that are likely to be contaminated. It has been hypothesized that the contamination of household drinking water may be influenced by several factors such as water storage and handling practices, domestic hygiene and surrounding environment, and social and cultural beliefs and practices [37]; and that improvement of drinking water quality alone might not be sufficient enough to reduce the incidence of diarrhea in places with poor environmental sanitation [38].

In this study the proportions of clinic visits and hospitalizations due to diarrhea and other illnesses were much higher than has previously been reported [27,39]. Although the high rate of clinic visits were expected because the field staff were instructed to encourage caregivers to bring their children to the study clinic whenever they were sick, the high hospitalization rate is possibly a more accurate estimation of the need for disease-related admissions among children residing in Indian slums than current hospitalization rates outside the setting of a study. A similar high rate of hospitalization and low mortality was also observed in the previous birth cohort study [9], which was attributed to good access to health care.

Per the National Family Health Survey 3 (NFHS-3) data, almost half of Indian children under the age of five years are stunted, about 20% are wasted and about 43% are underweight [40]. However, community-based studies have found a much higher prevalence of malnutrition among slum children in urban India [41]. In our study too, a large proportion of children were found to have one or more nutritional deficiencies during the follow-up period. It has been proposed that the high prevalence of childhood malnutrition in Indian slums is mainly due to factors such as inadequate food intake, recurrent illnesses and poor child care practices, as well as other issues such as lack of reach and coordination of public sector services, improper training and supervision of service providers, compromised efficiency of the nutritional programs and inadequate targeting of the urban poor [41].

As with other community-based studies, this study has several limitations. Most of the data on morbidities were collected through reporting by the primary caregiver. This could have resulted in over-reporting of certain morbidities that were perceived to be important by the caregivers and under-reporting of others considered not so important. Additionally, the fact that the children who were never breastfed were excluded from participation might have introduced an inherent selection bias, which in turn, could have resulted in underestimation of the true morbidity burden, since bottle-fed infants tend to have a higher risk of enteric and other infections [42-44]. However, we know from other observational studies in the same area that the proportion of children never breastfed is less than 3% (unpublished data).

Even though we provided sufficient quantities of bottled drinking water to cover the needs of the entire household by ensuring that water was available on demand, it was not possible to monitor or expect complete compliance. It is possible that children drank water from other sources or swallowed untreated water during bathing and other activities. In a recent study from an urban slum in Kolkata, India, it was found that water used for domestic purposes had a higher probability of contamination than that used for drinking purposes [45]. Also, no information was available on the quantity of water utilized for different household activities in our study. Studies have suggested that quantity of water available for domestic use, rather than the quality of source water might be a better predictor of diarrheal disease [46,47]. The lack of difference in GI illnesses between the bottled and municipal water cohorts could be explained partially by these limitations or by a high general level of environmental contamination which permits transmission of enteric infections through routes other than drinking water.

## Conclusions

This study demonstrated a continuing high burden of childhood illnesses among urban slum dwellers in southern India. On an average, a child was found to be ill for about three months in a year. In accordance with other studies in impoverished populations, most of the illnesses reported were of infectious origin. Frequent episodes of illness and the high degree of malnutrition experienced by children residing in this and similar settings may adversely impact their health and development, besides placing an additional burden on families who need to seek healthcare and find resources to manage disease. The apparent lack of a protective effect of drinking bottled water necessitates conduct of further studies to assess the effectiveness of alternate strategies, such as improvement of personal and peri-domestic hygiene, in reducing the burden of GI illnesses in settings with high environmental contamination.

## Competing interests

The authors declare that they have no competing interests.

## Authors’ contributions

RS coordinated the data collection, carried out the statistical analysis and drafted the manuscript. PS, BT and KNCS assisted with the study coordination and data management. JM, VB and ENN helped with the statistical analysis and in drafting of the manuscript. SSRA and HW participated in the design of the study and provided critical inputs in revising the manuscript. GK conceived the study, participated in its design and coordination and helped to draft and revise the manuscript. All authors read and approved the final manuscript.

## Pre-publication history

The pre-publication history for this paper can be accessed here:

http://www.biomedcentral.com/1471-2458/13/87/prepub
